# Elements of Medicine, &c.

**Published:** 1856-01

**Authors:** 


*Elements of Medicine, &c.* By S. H. Dicksen, M. D., L. L. D.

This is the title of a work recently issued by Blanchard & Lea, Phila., and is what its title denotes, "a comprehensive view" of the subjects of which it treats. He has his own peculiar views on the subject of contagion, in which he differs from the profession in the main; but with this exception, and one or two others of miner import, it is certainly a valuable contribution.

The style is easy and engaging, and no one who peruses it, will complain of its want of interest or life, and we are well assured that all who peruse it will not only be highly pleased, but largely profited.

